# A Case of Large Elbow Tuberous Xanthoma

**DOI:** 10.7759/cureus.39044

**Published:** 2023-05-15

**Authors:** Daniella Abramov, Tunisha Zaman, Ilya Tarascin

**Affiliations:** 1 Medicine and Surgery, The New York Institute of Technology College of Osteopathic Medicine, New York, USA; 2 Family Medicine, Nassau University Medical Center, New York, USA

**Keywords:** metabolism disorder, hyperlipidemia, lipoprotein, hypercholesterolemia, tuberous xanthomas

## Abstract

The purpose of this case report is to share the rare presentation of multiple giant tuberous xanthomas. Tuberous xanthomas are papulonodular skin lesions that are typically seen in patients with lipoprotein metabolism disorders. The patient in this report presented with large swellings on the right elbow and bilaterally on the Achilles tendons. Surgical excision of the mass in the right elbow revealed a tuberous xanthoma. Tuberous xanthomas are commonly seen in patients with lipid metabolism disorders which predisposes patients to developing morbid conditions. Therefore, while tuberous xanthomas are benign growths, patients should have a systemic workup performed in order to prevent or provide early intervention for morbid conditions.

## Introduction

Xanthomas are benign plaques, papules, or nodules that are composed of accumulated lipid-laden macrophages within the subcutaneous tissue [1]. Xanthomas can be differentiated into categories, with tendinous and tuberous xanthomas being the most common types in patients with a lipoprotein metabolism disorder, most commonly familial hypercholesterolemia [2]. Tendinous xanthomas are defined as subcutaneous tumors within tendons that facilitate the extension of extremities such as the extensor tendons of the buttocks and hands, as well as the Achilles and patellar tendons [3].

By contrast, tuberous xanthomas are less commonly seen and are defined as yellow nodules, usually less than 2 cm in diameter, located on the extensor aspects of the knees, elbows, and buttocks [3]. However, in this case, the histopathological examination revealed an atypically large tuberous xanthoma measuring about 5 cm in diameter. Other less common types of xanthomas that are seen in other metabolic disorders include eruptive, tuberoeruptive, or planar [3].

## Case presentation

**Figure 1:**
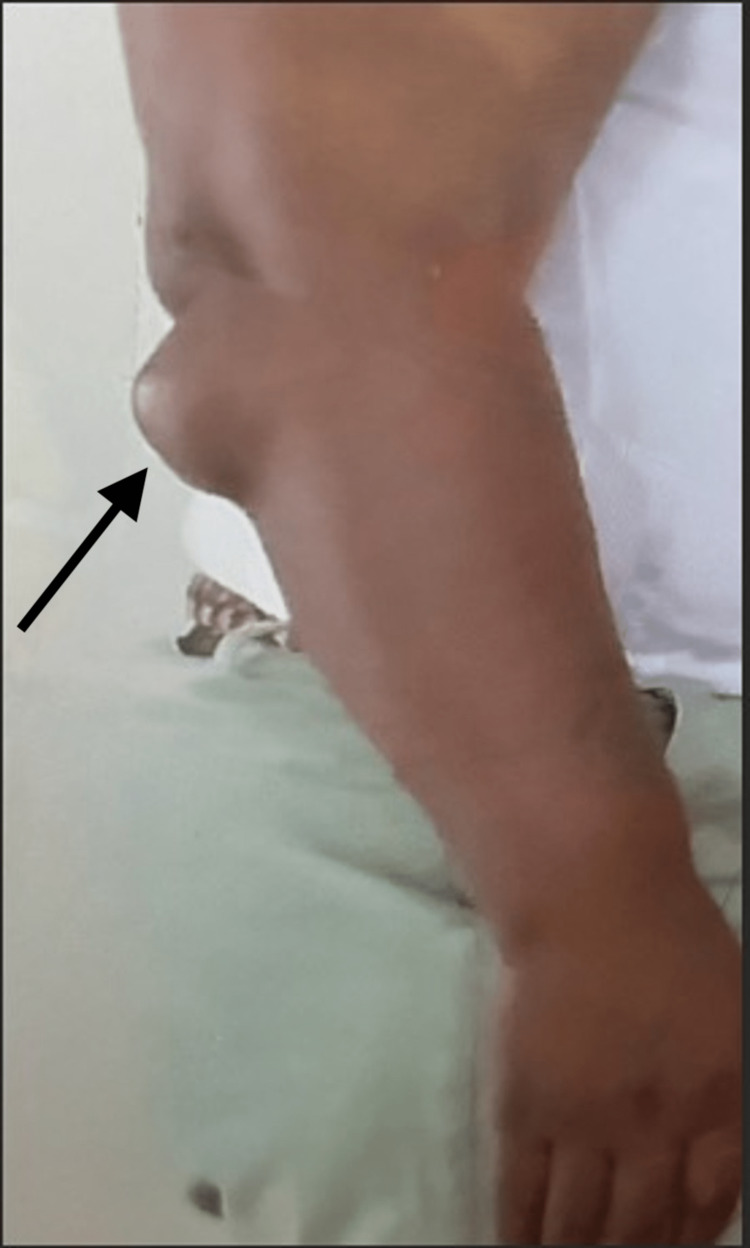
Large right elbow tuberous xanthoma

A 41-year-old female presented to the family medicine clinic with complaints of a right elbow mass that had been present over the course of several years and was beginning to limit her range of motion due to pain. On examination, the mass was firm, non-tender to palpation, mobile, without any skin changes, and measuring 5 x 5 cm (Figure 1). The remainder of the physical exam was unremarkable. She denied remarkable family history.

Upon initial and repeat review of the patient’s lipid profile, she was found to have elevated levels of low-density lipoproteins and total cholesterol. However, the triglycerides were within normal limits. Her lipid profile was as follows: total cholesterol: 239 mg/dL; low-density lipoprotein (LDL): 161 mg/dL; triglyceride (TG): 107 mg/dL; high-density lipoprotein (HDL): 57 mg/dL.

Ultrasound of the right elbow was performed with a high-frequency linear array transducer. It revealed a 5.0 x 1.7 x 5.4 cm heterogenous mass lesion of intermediate echogenicity in the subcutaneous soft tissue. There are a few non-shadowing echogenic foci internally along with areas of increased Doppler flow. The mass did not meet the criteria for a fluid collection of simple lipoma. Since the ultrasound findings were inconclusive, an MRI without contrast was recommended for further evaluation of the mass.

Due to the mass effects the patient was experiencing, she was referred to orthopedic surgery to remove the mass which was reviewed by a pathologist. The report indicated foam cells, also known as lipid-laden macrophages, which are indicative of tuberous xanthomas.

Upon further examination, the patient was found to have similar masses on the Achilles tendons bilaterally. The patient was advised to follow up with an MRI of the ankles bilaterally to confirm the presence of tuberous xanthomas over the affected areas; however, the MRI was denied on multiple occasions. The patient was not started on any medications at the family medicine clinic since she was referred to and received an upcoming appointment with an endocrinologist and lipidologist for genetic testing and preventative care related to her presentation. For the management of cholesterol, the lipidologist started the patient on oral atorvastatin 40 mg once per day. For weight loss, the endocrinologist started the patient on subcutaneous Ozempic 0.25 mg once per week which was titrated up to 0.50 mg per week after four weeks.

## Discussion

Xanthomas are palpable masses that are characterized by the accumulation of lipid-laden macrophages within the subcutaneous tissue [1]. They most commonly manifest in patients with a lipid metabolism disorder. Tuberous xanthomas classically appear as red to yellow papulonodular lesions of the skin found over the extensor surfaces of the knees, elbows, and buttocks [3]. It has been suggested that extensor surfaces are most commonly affected due to mechanical stress. Mechanical stress of extensor tendons exposes these areas to minor but recurrent trauma which is believed to increase capillary permeability to lipids and potentially contribute to the predisposition of these areas to develop tuberous xanthomas [4].

As previously stated, tuberous xanthomas are typically less than 2 cm in diameter. Therefore, the presentation of our patient with a tuberous xanthoma that was 5 cm in diameter is atypical. Upon initial evaluation, a tuberous xanthoma must be differentiated from a tumor which can be done with imaging. Ultrasound has been demonstrated as an effective form of imaging for identifying xanthomas [5]. In our case, ultrasound was not able to provide a definitive diagnosis, prompting further evaluation with MRI. MRI scan is another useful imaging tool to differentiate xanthomas from tumors. Xanthomas on tendons have increased signal intensity on T1 and T2; however, tumors typically have much greater signal intensities compared to xanthomas [6].

Once a patient is diagnosed with a tuberous xanthoma, it is essential to complete a systemic workup in order to prevent or provide early intervention for morbid conditions such as familial hypercholesterolemia. Workup consists of fasting laboratory tests of plasma TG, plasma cholesterol, serum LDL, and HDL [3]. These patients should have a close follow-up in order to decrease complications related to cardiovascular consequences such as atherosclerosis and coronary artery disease [3]. Medical management with HMG-CoA reductase inhibitors has been reported to potentially reduce the size of xanthomatous lesions [7]. The patient in this case was started on atorvastatin, which is an HMG-CoA reductase inhibitor that works by blocking the rate-limiting step of cholesterol synthesis. In this case, the patient was treated with surgical intervention due to the limited range of motion from the mass effects. Surgical intervention is not recommended unless the xanthoma is associated with pain or limited range of motion. However, it has been reported that postsurgical recurrence of xanthomas is high but can be avoided with HMG-CoA reductase inhibitors [8].

## Conclusions

Fundamentally, tuberous xanthomas are palpable masses that are characterized by the accumulation of lipid-laden macrophages within the subcutaneous tissue which are most commonly seen in patients with lipid metabolism disorders. Ultrasound can contribute to the diagnosis; however, it is not a diagnostic method for xanthomas. MRI and US can improve the accuracy in identifying xanthomas, but a biopsy is required for confirmation when deemed necessary. Patients with a diagnosis of tuberous xanthoma should have a systemic workup performed in order to prevent or provide early intervention for morbid conditions. Interventions with medications such as HMG-CoA reductase inhibitors have reportedly reduced the size of xanthomatous lesions, but surgical intervention may be required in patients who experience pain or limited range of motion due to mass effects.
